# Cardiac troponin T in extracellular vesicles as a novel biomarker in human cardiovascular disease

**DOI:** 10.1002/ctm2.979

**Published:** 2022-08-21

**Authors:** Kathleen M. Lennon, Andras Saftics, Sarah Abuelreich, Parul Sahu, H. Immo Lehmann, Adam L. Maddox, Reem Bagabas, James L. Januzzi, Kendall Van Keuren‐Jensen, Ravi Shah, Saumya Das, Tijana Jovanovic‐Talisman

**Affiliations:** ^1^ Department of Molecular Medicine Beckman Research Institute of the City of Hope Comprehensive Cancer Center Duarte CA USA; ^2^ Cardiovascular Research Center Massachusetts General Hospital and Harvard Medical School Boston MA USA; ^3^ Neurogenomics Division Center for Noninvasive Diagnostics Translational Genomics Research Institute Phoenix AZ USA; ^4^ Department of Molecular and Cellular Biology Beckman Research Institute of the City of Hope Comprehensive Cancer Center Duarte CA USA

Dear Editor,

Soluble cardiac troponin T (cTnT), an indicator of myocardial injury and stress, is used in decision management for patients with cardiovascular disease (CVD). As highly sensitive assays can detect elevated concentrations of cTnT even in healthy individuals (e.g. outside of myocardial necrosis, electrocardiographic changes or angina) and cannot distinguish among disease conditions,^1^, ^2^ a comprehensive understanding of the cTnT‐secretome is an unmet need.

Within the secretome, cTnT is not only present as a soluble factor but may also be contained within extracellular vesicles (EVs).^3^ EVs are nanoscale particles secreted by all cells, the cargoes of which can reflect the molecular composition of the cells of origin^4^ and indicate disease or injury.^5^ As EVs are easily sampled from plasma,^6^ they are being developed as a ‘liquid’ biopsy reflecting the disease state from the tissue of origin. Here, we advanced a fluorescence‐based super‐resolution microscopy technique, quantitative single‐molecule localization microscopy (qSMLM), to robustly characterize cTnT‐positive EVs. Importantly, we provide the first report of cTnT‐secretome across a spectrum of CVDs.

EVs were purified from induced pluripotent stem cell–derived cardiomyocyte cell media (CCM), representing a source of cardiomyocyte‐derived EVs (Figure S1), and patient plasma (Figures S2): healthy subjects (*n* = 5), patients with heart failure (HF; *n* = 5), hypertrophic cardiomyopathy (*n* = 3), type 1 myocardial infarction (MI‐TI, *n* = 5) or type 2 myocardial infarction (MI‐TII; *n* = 5) and chronic kidney disease (CKD; *n* = 5). In all cases (Figures S1 and S2), EVs had intact morphology and contained canonical EV markers (tetraspanins CD9, CD63, CD81; luminal marker TSG101) with low amounts of soluble proteins. According to dot blots (Figure S2C), the CD81 content of patient EVs was highly variable, whereas combined tetraspanins had more uniform expression. Table S1 shows patient characteristics. Control patients were younger and more likely to be female. Patients with MI and CKD had a higher incidence of diabetes and CAD, whereas HF patients had lower mean left ventricular ejection fraction and higher NT‐proBNP.

To enable molecular quantification of EVs using qSMLM, the following five steps were performed (see the Supplemental Methods): (1) covalent labelling of membrane proteins with fluorescent dye CF568 to detect EV membranes; (2) affinity labelling of cTnT with a specific AF647‐tagged antibody (in mild permeabilizing conditions) to detect cTnT‐EV cargo; (3) affinity labelling of EVs with unmodified tetraspanin antibodies to isolate tetraspanin‐enriched EVs onto coverslips; (4) two‐colour imaging to detect EV membrane and cTnT; and (5) data analysis^7^ with robust molecular counting (Figure S3)^8^ to quantify images. We first validated the assay and assessed CD81‐enriched CCM‐EVs (Figure S4). Overall, ∼14% of EVs contained cTnT; on average, these EVs had a diameter of 118 nm with two detected molecules of cTnT per EV (Figure S4D,E). Next, we affinity isolated tetraspanin‐enriched plasma EVs (using a combination of antibodies against the canonical EV markers CD81, CD63 and CD9 for affinity pull‐down). EV membrane and cTnT were fluorescently labelled and detected using qSMLM (Figure 1A). Characterizations of EVs for individual subjects and controls are provided in Figures S5 and S6. The percentage of cTnT‐positive EVs did not vary significantly across CVDs (Figure 1B). Although the overall size distribution of plasma EVs was similar across all CVDs (Figure 1C, left), cTnT‐positive EVs were on average significantly larger (Figure 1C, right) with a typically narrower range of sizes (Figure 1D). This was in agreement with CCM‐EVs (Figure S4D,E): cTnT‐positive EVs had a larger average diameter with a narrower range of sizes. Importantly, cTnT‐positive EVs in MI‐TI, MI‐TII and CKD (but not in HF) were smaller than those in healthy donors; the difference was significant both when EVs were averaged per subject (Figure 1C, right) and when EVs from subjects with different diagnoses were grouped (Figure 1E). This raised the possibility that the biogenesis of cTnT‐EVs may differ across CVDs.

**FIGURE 1:**
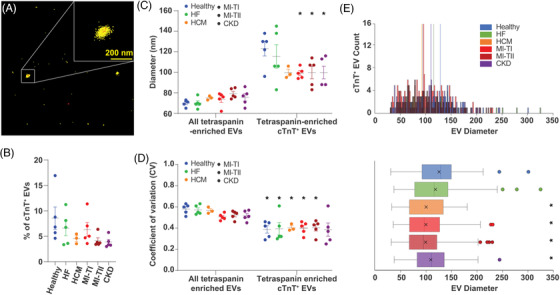
Size quantification of extracellular vesicles (EVs) from healthy subjects and patient plasma: (A) representative quantitative single‐molecule localization microscopy (qSMLM) image of tetraspanin‐enriched EVs from patient plasma with CF568 localizations in yellow (detection of EV membrane) and cardiac troponin T (cTnT) Ab‐AF647 localizations in red (detection of cTnT); cTnT‐positive EV is shown in the inset. (B) Percentage of cTnT‐positive EVs (averaged per subject) within the total tetraspanin‐enriched EV populations. Across healthy subjects and different disease conditions, ∼5% of EVs were cTnT‐positive. (C) EV diameter (averaged per subject) for all tetraspanin‐enriched EVs and tetraspanin‐enriched, cTnT‐positive EVs is reported in healthy subjects and different disease conditions. Compared to all tetraspanin‐enriched EVs, tetraspanin‐enriched, cTnT‐positive EVs had larger diameter. Compared to healthy individuals, type 1 myocardial infarction (MI‐TI), type 2 myocardial infarction (MI‐TII) and chronic kidney disease (CKD) patients had tetraspanin‐enriched, cTnT‐positive EVs with significantly smaller diameter; there was no size difference for healthy subjects and heart failure (HF) patients. (D) The cTnT‐positive EVs typically had smaller coefficients of variation compared to the total tetraspanin‐enriched EV population. (E) EVs from subjects with different diagnoses were grouped. Histograms of distributions for EV size are shown on top, whereas corresponding box plots are on the bottom. Box plots represent the interquartile range, median (centre line) and mean (indicated with x); dots indicate EVs beyond 1.5 times the interquartile range. * indicates *p* < .05. All individual samples had at least two independent replicates, minimum 15 FOV. For B–D, error bars represent SEM.

Using a highly sensitive clinical assay, we measured soluble cTnT in circulation (‘hs‐cTnT’). Individuals with MI‐TI had the highest hs‐cTnT values followed by MI‐TII and CKD (Figure 2A, Table S1). qSMLM provided complementary information on cTnT content within purified EVs (number of detected cTnT molecules per EV). Interestingly, qSMLM for MI and CKD patients (compared to healthy or HF) detected a significantly lower average amount of cTnT per EV (Figure 2B,C). Consequently, in samples with elevated levels of hs‐cTnT, qSMLM revealed a smaller EV diameter and reduced content of cTnT per EV (Figure 2D,E). No differences were observed by qSMLM between females and males for both EV size and cTnT per EV; only small variations were observed in EV size with body mass index (Figure S7). This study reveals a striking discordance between soluble clinical hs‐cTnT in plasma and the number of qSMLM detected cTnT molecules per EV across CVDs.

**FIGURE 2:**
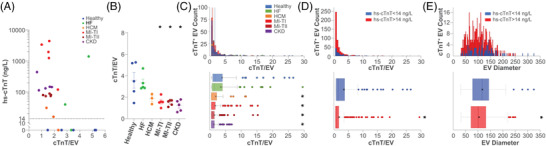
Characterization of tetraspanin‐enriched, cardiac troponin T (cTnT)‐positive extracellular vesicles (EVs) across clinical troponin measurements: (A) average number of detected cTnT molecules per EV for each patient as a function of clinical hs‐cTnT measurements; each dot represents a subject. The dashed line represents clinically relevant soluble hs‐cTnT cut‐off. (B) Across healthy subjects and different disease conditions, the number of detected cTnT molecules per EV (averaged per subject) is reported. Compared to healthy individuals, type 1 myocardial infarction (MI‐TI), type 2 myocardial infarction (MI‐TII) and chronic kidney disease (CKD) patients had EVs with significantly lower cTnT per EV; there was no difference in cTnT per EV for healthy subjects and heart failure (HF) patients. All individual samples had at least two independent replicates, minimum 15 FOV. All error bars represent SEM. (C) EVs from subjects with different diagnoses were grouped. Histograms of distributions for detected cTnT molecules per EV are on top, whereas corresponding box plots are on the bottom. (D and E) EVs from subjects with positive (>14 ng/L in red) and negative (<14 ng/L in blue) clinical hs‐cTnT values were grouped. Histograms of distributions for detected cTnT per EV (D) and EV size (E) are on top, whereas corresponding box plots are on the bottom. Compared to subjects with negative clinical hs‐cTnT content, subjects with positive clinical hs‐cTnT had EVs with significantly lower value of cTnT per EV and smaller size. (C–E) Box plots represent the interquartile range, median (centre line) and mean (indicated with x); dots indicate EVs beyond 1.5 times the interquartile range. * indicates *p* < .05.

Although our sample size was small (due to the work‐intensive nature of qSMLM), we comprehensively characterized cTnT‐positive EVs. Our findings are consistent with a prior study that detected cTnT in large EVs from infarcted mice hearts and patients undergoing cardiopulmonary bypass.^3^ Additionally, our findings point to potentially distinct mechanisms of origin for circulating cTnT across the range of CVDs. Although MI leads to myonecrosis, which yields free cTnT in plasma, changes in wall stress in HF patients appeared to precede changes in release of cTnT, suggesting a potentially different mechanism for release of circulating cTnT in HF patients,^9^ such as the release of cTnT‐EVs. A unique innovation of our approach is the molecular assessment of individual cTnT‐positive EVs. These results may also provide context for why cardiac troponin detection in patients treated with sarcomeric modulators is not necessarily related to myonecrosis or poorer outcomes.^1^, ^10^

In conclusion, the molecular profile of individual EVs offers important biological insight into cardiomyocyte biology and refines the measurement of established biomarkers. Our study demonstrates the presence of cTnT within EVs derived from cardiomyocytes in human subjects, including healthy controls. Further, our technology differentiated the biophysical characteristics and cTnT content of EVs across different CVDs. qSMLM data captures how cTnT‐positive EVs differed among patients with different causes and severity of cardiac injury, providing complementary information to clinical hs‐cTnT to more comprehensively describe the cardiac secretome. Future studies to determine the prognostic implications of cTnT‐EVs are warranted.

## CONFLICT OF INTEREST

RS has served as a consultant for MyoKardia (concluded 2/28/2021), Cytokinetics (ongoing) and Best Doctors (completed 6/2021) and has been on a scientific advisory board for Amgen (ongoing). RS is a co‐inventor on a patent for ex‐RNAs signatures of cardiac remodelling. KVJ is a member of the Scientific Advisory Board for HTG and Dyrnamix, neither of which has played a role in this study. SD is a founding member and holds equity in LQTT and Switch Therapeutics and has consulted for Renovacor, none of which played any role in this study.

## Supporting information

Supporting Information
