# Supplementary material for: Cardiac troponin T in extracellular vesicles as a novel biomarker in human cardiovascular disease
Source: Clin Transl Med. 2022 Aug 21;12(8):e979. doi: 10.1002/ctm2.979 (PMC9393073; doi:10.1002/ctm2.979)
Supplement: Supplementary file 1 — Supporting Information [file CTM2-12-e979-s001.docx]

**Supplemental Methods**

**Cell Culture.** To generate cardiomyocyte conditioned media, iPSCs (BJ RiPS-E; RRID:CVCL_X739) were first differentiated into cardiomyocytes. iPSCs were cultured with Complete Gibco™ Essential 8™ Media with Supplements, 10 μM Y inhibitor (Stemgent; 04-0012-10) for three days. 95% confluent cells were treated with 10 μM CHIR99021 (Stemgent; 04-0004) in RPMI 1640 Medium (Thermo Scientific; 61870127) with B27 Supplement without insulin (Thermo Scientific; A1895601). This was considered Day 0. On day 3, cells were treated with 5 μM IWP4 Stemolecule Wnt Inhibitor IWP-4 (Stemgent; 04-0036) in RPMI 1640 medium including B27 Supplement without insulin. On day 7, cells were fed with B27 Supplement with insulin (Thermo Scientific; 17504044) in RPMI 1640. The differentiated, cardiomyocyte cells were kept on metabolic selection starting on day 10 for six days using 1X Sodium lactate (Sigma-Aldrich; L7022-10G) in RPMI 1640 Medium, no glucose (Thermo Scientific; 11879020). On Day 16, cells were trypsinized (TrypLE™ Express Enzyme (1X), Thermo Scientific; 12605028) and plated on six-well plates coated with Geltrex™ LDEV-Free, hESC-Qualified, Reduced Growth Factor Basement Membrane Matrix (Thermo Scientific; A1413302) with RPMI 1640 supplemented with 10% FBS. Characterization of iPSC-CMs demonstrated the presence of known validated markers of CM differentiation by immunostaining (Figure S1A). On day 20, cell media was changed to RPMI 1640 with B27 supplement and insulin (serum-free media). Conditioned media was collected after 48 hours for EV isolation (day 22). The collected supernatant was spun at 2000g for 10 minutes and passed through a 0.8 μm syringe filter.

**Purification of EVs from Cell Media.** EVs were isolated and purified as previously described^1^. Briefly, clarified conditioned media was concentrated to 400 μL using a Vivaspin 20 100 kDa concentrator (VWR; 95056-134) by centrifuging at 1000g in 10 minute increments. The resulting 400 μL of concentrated media was loaded onto a qEV_original_ 70 nm column (iZON) equilibrated at room temperature with PBS, according to manufacturer instructions. SEC was carried out with the qEV IZON automatic fraction collector, which begins EV fraction collection after the discarded void volume. 3 mL of void volume is discarded into the waste reservoir then 500 μL fractions 1-13 were collected from the qEV_original_ on the iZON fraction collector (FC). FC fractions (FCF) were analyzed by NTA for EV concentration and by NanoDrop for protein concentration. FCF1-4 were combined and concentrated using an Amicon Ultra centrifugal 100 kDa concentrator (Millipore Sigma; UFC210024) by centrifuging at 1000g for 3-5 minutes. Amicon concentrators were pre-incubated with 0.05% IgG-Free BSA (Jackson ImmunoResearch; 001-000-162) for 30 minutes at room temperature to minimize EV loss on the membrane.

**Subject Inclusion.** For EV purification from plasma, several cohorts of subjects were included; healthy subjects (n=5), patients with heart failure (HF, n=5), hypertrophic cardiomyopathy (HCM, n=3), type 1 myocardial infarction (MI-TI, n=5) type 2 myocardial infarction (MI-TII, n=5), and chronic kidney disease (CKD, n=5). MI was defined using the 4^th^ Universal Definition of MI^2^: A fifth-generation high sensitivity cardiac troponin T concentration (measured with Elecsys Troponin T Gen 5 STAT) of ≥10 ng/L for women, or ≥15 ng/L for men, were diagnostic of myocardial injury An MI was defined as a rising and/or falling elevation in high-sensitivity cardiac troponin (hs-cTnT) >99th percentile and at least one of the following: 1) symptoms of ischemia, 2) new electrocardiographic evidence of ischemia, 3) new pathological Q waves, 4) new regional wall motions on imaging in an ischemic territory, or 5) coronary thrombus on angiography^2, 3^. Type II MI was diagnosed in those patients, where no coronary trigger was present^4^. All cases were reviewed by study staff and checked by H.I.L prior to inclusion into this study to determine adjudication of the correct respective diagnosis.

**EV Purification from Plasma.** At first, 400 μL of plasma was loaded onto an equilibrated qEV_original_ 70 nm column. 500 μL fractions 1-13 were collected from the iZON fraction collector. FCF1-4 were pooled together. Initially, pFCF1-4 were analyzed by NTA to determine EV concentration and NanoDrop to measure protein concentration/assess purity. The assessed patient samples had consistent results. Therefore, we used the same volume (150 μL) of EVs for qSMLM experiments.

**Nanoparticle Tracking Analysis (NTA) and Negative Staining Transmission Electron Microscopy (TEM)** were performed as before^1^ on pFCF1-4.

**Dot Blot.** pFCF1-4 EVs were dotted onto nitrocellulose membranes and allowed to dry as previously described^5^. Manufacturer protocol was followed. Antibodies used for these experiments were anti-TSG101 (1:1000, Sigma; T5701-200UL), anti-CD9 (1:1000, Biolegend; 312102), anti-CD63 (1:1000, Novus; NBP2-42225), and anti-CD81 (1:1000, Biolegend; 349502), goat anti-rabbit IRDye 680RD (1:15000, LI-COR) or goat anti-mouse IRDye 800CW (1:15000, LI-COR). Fluorescent antibody detection was performed using the LI-COR Odyssey CLx imaging system. Sypro Ruby was used to detect the total protein amount, per manufacturer instructions. Sypro Ruby stained membranes were imaged on a Bio-Rad Chemidoc imaging system.

**Antibodies.** The following molecules, primary, and secondary antibodies were used for the detection and affinity isolation of EVs: maleimide-CF568 (Sigma-Aldrich; SCJ4600026-1UMOL), anti-CD9 (1:1000, Biolegend; 312102), anti-CD63 (1:1000, Novus; NBP2-42225), anti-CD81 (1:1000, Biolegend; 349502), anti-cTnT (Abcam; ab8295), goat anti-mouse (Millipore; Ap124), and highly cross-adsorbed goat anti-rabbit IgG (Invitrogen; A16112).

The cTnT primary antibody was fluorescently labeled with Alexa Fluor 647 (AF647) NHS (Thermo Scientific; A20006), as previously described^6^. Briefly, 50 µL of 1 mg/mL antibody in PBS supplemented with 2 mM of sodium bicarbonate was incubated with 4 times molar excess of AF647 NHS (30 minutes at room temperature). After quenching with 150 mM hydroxylamine HCl pH 8.5, unbound dye molecules were removed using Micro bio-spin 6 gel filtration columns (Bio-Rad) equilibrated with PBS, and any remaining aggregates were removed with Nanosep 300kD filters (PALL). The degree of labeling was determined with NanoDrop; the values ranged between 1 and 2 dyes per antibody.

To characterize the photophysical properties of fluorescently labeled cTnT Ab, we followed previously published protocol^7^. Surfaces with sparse, covalently attached cTnT Ab-AF647 were imaged under the same conditions as surfaces with EVs, as described below. The average number of localizations for the fluorescent reporter (cTnT Ab-AF647) was determined to be 4 (Figure S3A,B).

**Labeling of EVs from iPSC-cardiomyocyte cell media (CCM)**. Maleimide-CF568 (mal-CF568) stock was prepared in DMSO, per manufacturer instructions. 7×10^8^ EVs from CCM were diluted in EV blocking buffer (PBS with 0.5% BSA and 0.01% Tween-20) to a final volume of 150 μL. 15 nmol of mal-CF568 was added to the 150 μL EV sample, protected from light, and rotated for 1 hour at room temperature. Maleimide labeling was quenched with 5 times molar excess L-glutathione (Sigma; 101179609) for 10 minutes. Excess mal-CF568 was removed through purification with a qEV_single_ 70 nm iZON column. The 150 μL mixture of labeled EVs was loaded onto a PBS equilibrated qEV_single_ column, 850 μL of flow through was discarded and three 200 μL fractions containing EVs were collected and concentrated in a 100 kDa concentrator (Millipore Sigma; UFC210024). To reduce the loss of EVs on the concentrator membrane, 100 kDa concentrators were pre-incubated with 0.05% IgG-free BSA (Jackson ImmunoResearch; 001-000-162). EVs were concentrated to less than 150 μL for incubation with primary antibodies. Primary antibodies were prepared in a 50 μL permeabilization blocking buffer such that the final EV buffer would be 0.5% BSA, 0.05% Tween-20 in PBS, 50 nM cTnT Ab-AF647, and 15 nM anti-CD81 Ab. The EV solution was protected from light and rotated for 45 minutes at room temperature. EVs were fixed with 16% (w/v) paraformaldehyde stock (Electron Microscopy Sciences; 15710) for 30 minutes (final concentration of fixative was 4%), then quenched with 25 mM glycine. EVs were concentrated in a 100 kDa concentrator (pre-incubated with IgG-free BSA, as described above) to 200 μL. Excess primary antibody was removed through a qEV_single_ 70 nm iZON column purification: the 200 μL mixture of labeled EVs with primary antibody was loaded onto a PBS equilibrated qEV_single_ column, 800 μL of flow through was discarded and three 200 μL fractions containing EVs were collected into a 100 kDa concentrator (pre-incubated with IgG-free BSA, as described above). The resulting 600 μL of labeled EV containing fractions were concentrated to less than 200 μL by centrifugation (1000g, 5 minutes) for incubation on surfaces coated with secondary Abs.

**Labeling of EVs from plasma.** 150 μL of pFCF1-4 EVs from plasma were incubated with 15 nmol of mal-CF568, protected from light, and rotated for 1 hour at room temperature. Maleimide labeling was quenched with 5 times molar excess L-glutathione (Sigma; 101179609) for 10 minutes. Excess mal-CF568 was removed by centrifugation in a 100 kDa concentrator (Millipore Sigma; UFC210024) pre-incubated with IgG-free BSA. In the 100 kDa concentrators, EV samples were washed with 1 mL of blocking buffer and centrifuged (1000g) for 5 minutes (repeated three times). EVs were concentrated to less than 150 μL for incubation with primary antibodies. Primary antibodies were prepared in 50 μL permeabilization blocking buffer such that the final EV buffer would be 0.5% BSA, 0.05% Tween-20 in PBS, 50 nM cTnT Ab-AF647, 15 nM anti-CD81, 1.5 nM anti-CD9, and 7.5 nM anti-CD63. The EV solutions were protected from light and rotated for 45 minutes at room temperature. Excess primary antibodies were removed through a qEV_single_ 70 nm iZON column purification: The 200 μL mixture of labeled EVs was loaded onto an equilibrated qEV_single_ column and processed as before. The resulting 600 μL of EV containing fractions were concentrated to less than 200 μL by centrifugation (1000g, 5 minutes) for incubation on surfaces coated with secondary Abs.

**Affinity isolation of EVs from CCM or plasma onto coverslips**. Secondary antibody was covalently attached to the activated coverslips as previously described^1, 7^. Labeled EVs were incubated on mouse secondary antibody or rabbit secondary antibody (control) coated surfaces, as indicated, for 1 hour at room temperature. Surfaces were washed with EV blocking buffer and PBS, fixed with 4% paraformaldehyde (VWR) and 0.2% glutaraldehyde (VWR) for 30 minutes, and quenched for 10 minutes with 25 mM glycine.

**dSTORM Imaging.** Surfaces placed into the Attofluor cell chamber (Life Technologies) in dSTORM imaging buffer^8^ were imaged immediately after preparation. Imaging was performed on a 3D N-STORM super-resolution microscope (Nikon). Ti2-E inverted microscope with a piezo stage and Perfect Focus System included a 100× 1.49 NA total internal reflection (TIRF) objective (Apo), N-STORM lens, λ/4 plate, a Quad cube C-NSTORM (97355 Chroma), and a 2× magnification lens. A Nikon LUNF commercial launch included 405, 488, 561, and 647 nm lasers. Images are captured with an EM-CCD camera iXon DU897-Ultra (Andor Technology).

EVs were located in TIRF illumination using fluorescent signal from the membrane labeled with CF568, with a low 561 nm laser power. Then, dSTORM images of 41 × 41 μm were collected with an exposure time of 10 ms using NIS-Elements 5.21.01 Software (Nikon). 10,000 frames were acquired for each field of view (FOV), in each channel. To excite AF647 labeled primary antibodies, the 647 nm laser power was set to 89.1 mW. Subsequently, CF568 was activated/excited using the 561 nm laser with the power set to 12.8-15.8 mW. 647 nm and 561 nm acquisitions were collected separately, using the appropriate emission filters.

**Photophysical characterization of a fluorescent reporter**. To assess cTnT in EVs with qSMLM, we used a well characterized monoclonal Ab^9^ labeled with Alexa Fluor 647 (AF647). Our labeling protocol resulted in an average of approximately one dye molecule per one antibody molecule (degree of labeling approximately 1, detailed in Methods and ^1^). To enable the quantitative analysis of SMLM images, we characterized the photophysical properties of this fluorescently labeled reporter. In particular, we used a surface assay for molecular isolation (SAMI)^7^ to determine the average number of localizations from AF647 labeled anti-cTnT antibody (cTnT Ab-AF647) (Figure S3A,B). We have previously shown^1^ that this approach enables the robust quantification of detected molecules on EVs with qSMLM.

**Multi-channel image alignment.** Multi-channel alignment was performed with images of fluorescent beads used as fiducial markers (TetraSpeck 0.1 µm microspheres from Thermo Scientific; T7279). Beads randomly distributed on poly-L-lysine-coated glass coverslips were imaged in the described qSMLM setup in both 647 nm and 561 nm channels. The position of each bead center was acquired from bead localizations using a custom-written MATLAB code. An ensemble image of bead center coordinates obtained from multiple FOV was generated for 561 nm and 647 nm channels, considering only centers which appeared in both channels. Bead center coordinates in the 561 nm channel were used as a set of control points for inferring a spatial transformation that allows for overlaying bead centers from the 647 nm channel (moving points). Spatial transformation was performed using a local weighted mean algorithm of the built-in MATLAB function *cp2tform*, generating a 2^nd^ order polynomial transformation function, as described elsewhere^11-13^. The obtained transformation function was then applied to each multicolor region to align the 647 nm and 561 nm channels (Figure S3C,D).

**Data Analysis.** Using NIS-Elements, peaks of the detected and processed photon counts originating from excited fluorophores with a minimum height above 3500 (a.u.) for the 561 nm channel and 5000 (a.u.) for the 647 nm channel were fit from raw image data. We performed a multi-channel alignment (described in detail above) then overlayed sequential images of the same FOV, in the separate channels. Voronoi tessellation was used to segment localizations from the 561 nm channel into “clusters” and extract EV diameters. Small and large EV “clusters” had different tessellation thresholds to account for differences within localization patterns. EVs with a radius below 15 nm or with fewer than 15 localizations were removed, EVs with more than 2000 (for “small” EVs with below 75 nm radii) or 15000 (for “large” EVs with greater than 75 nm radii) localizations were excluded as these puncta represent artifacts with persistent fluorescence rather than biologically relevant signal from blinking molecules. Tessellation polygons were considered clustered where each polygon area was smaller than 600 nm^2^. The Voronoi tessellation method was based on the ClusterViSu algorithm^10^ with modifications to improve processing speed (rapid Voronoi tessellation)^1^. Following the determination of EVs based on Voronoi clusters, the radius of clustered EV regions plus an additional 50 nm was assessed for a 647 nm signal (colocalization) (Figure S3E), encompassing the total region of the EV plus a border allowing for any chromatic aberration error. Localizations in the 647 nm channel which appeared in 200 consecutive frames within a radius of 24 nm (maximal localization precision value × 2) were grouped, and 647 nm channel localizations were assessed for molecular content using obtained alpha value^7^. To ensure detected cTnT content of vesicles was biophysically relevant, the maximal number of possible cTnT molecules in an EV within a given diameter was calculated by dividing the spherical volume of a detected EV by the estimated spherical volume of cTnT protein bound to a 150 kD antibody. Colocalized signals above this threshold were excluded. Additionally, colocalized 647 nm signal of more than 120 localizations was considered an imaging artifact and was not considered to be cTnT-positive EVs. Of note, only one EV out of the total colocalized EVs from all patient samples was excluded using this threshold.

**Patient Cohort.** Baseline characteristics of the patient population are depicted in Table S1. The qSMLM team was blinded to patient status and clinical cTnT levels until all data acquisition and analyses were completed. All patients provided informed consent before EDTA plasma samples and baseline characteristics were obtained. This study was approved by the IRB of the Massachusetts General Hospital, Boston, USA (IRB numbers 2017P002010, 2016P001250, 2017P002010 (for HCM), and 2017P000220).

**Statistical Information.** All cell culture EV experiments were run in triplicates using separate cell culture preparations (biological replicates), with five FOV (technical replicates). Due to limited sample volume, each patient and healthy subject sample were run at least in duplicate, with a minimum of 7 FOV per run (total FOV at least 15). If few EVs were detected in a FOV, more fields were imaged to obtain statically relevant information. Mean and standard error of the mean (SEM) values were determined using GraphPad Prism, Microsoft Excel, and MATLAB software packages. The coefficient of variation (CV) was used to describe the variance within EV populations. Scatter plots, histograms, as well as box and whisker plots were generated in MATLAB, and graphs were made using GraphPad Prism Software. Two tails student t-test (p-value) were used to determine whether the two populations were significantly different.

1. Lennon, K.M.*, et al*. Single molecule characterization of individual extracellular vesicles from pancreatic cancer. *Journal of extracellular vesicles*. **8**, 1685634 (2019).

2. Thygesen, K.*, et al*. Fourth Universal Definition of Myocardial Infarction (2018). *Circulation*. **138**, e618-e651 (2018).

3. McCarthy, C.P.*, et al*. The Intersection of Type 2 Myocardial Infarction and Heart Failure. *J Am Heart Assoc*. **10**, e020849-e020849 (2021).

4. Sandoval, Y. & Jaffe, A.S. Type 2 Myocardial Infarction: JACC Review Topic of the Week. *Journal of the American College of Cardiology*. **73**, 1846-1860 (2019).

5. Nizamudeen, Z.*, et al*. Rapid and accurate analysis of stem cell-derived extracellular vesicles with super resolution microscopy and live imaging. *Biochimica et biophysica acta. Molecular cell research*. **1865**, 1891-1900 (2018).

6. Tobin, S.J.*, et al*. Single molecule localization microscopy coupled with touch preparation for the quantification of trastuzumab-bound HER2. *Scientific reports*. **8**, 15154-15154 (2018).

7. Golfetto, O.*, et al*. A Platform To Enhance Quantitative Single Molecule Localization Microscopy. *J. Am. Chem. Soc.* **140**, 12785-12797 (2018).

8. Dempsey, G.T., Vaughan, J.C., Chen, K.H., Bates, M. & Zhuang, X.W. Evaluation of fluorophores for optimal performance in localization-based super-resolution imaging. *Nat. Methods*. **8**, 1027-1036 (2011).

9. Gladka, M.M.*, et al*. Cardiomyocytes stimulate angiogenesis after ischemic injury in a ZEB2-dependent manner. *Nature communications*. **12**, 84 (2021).

10. Andronov, L., Orlov, I., Lutz, Y., Vonesch, J.L. & Klaholz, B.P. ClusterViSu, a method for clustering of protein complexes by Voronoi tessellation in super-resolution microscopy. *Sci Rep*. **6**, 24084 (2016).

11. Churchman, L.S. & Spudich, J.A. Colocalization of fluorescent probes: accurate and precise registration with nanometer resolution. *Cold Spring Harbor protocols*. **2012**, 141-149 (2012).

12. Churchman, L.S. & Spudich, J.A. Single-molecule high-resolution colocalization of single probes. *Cold Spring Harbor protocols*. **2012**, 242-245 (2012).

13. Erdelyi, M.*, et al*. Correcting chromatic offset in multicolor super-resolution localization microscopy. *Opt Express*. **21**, 10978-10988 (2013).

**Supplemental Table**

|  | **All**  **(n=28)** | **Control Patients (n=5)** | **HF**  **(n=5)** | **HCM**  **(n=3)** | **MI-TI**  **(n=5)** | **MI-TII**  **(n=5)** | **CKD**  **(n=5)** |
| --- | --- | --- | --- | --- | --- | --- | --- |
| **Age, y** | 63.7±13.7 | 44.8±10.3 | 71.6±12.7 | 64±17 | 66±4 | 73±7 | 63±13 |
| **Female sex, n (%)** | 15/28 (54) | 4/5 (80) | 2/5 (40) | 2/3 (40) | 2/5 (40) | 2/5 (40) | 2/5 (40) |
| **BMI, kg/m²** | 27.4±5.0 | 31.8±3.9 | 26.9±7.1 | 24.7±5.4 | 25.4±2.4 | 27.8±6.1 | 26.8±3.0 |
| **Diabetes, n (%)** | 12/28 (43) | 0/5 | 1/5 (20) | 1/3 (20) | 3/5 (60) | 3/5 | 4/5 (80) |
| **Hx of smoking, n (%)** | 8/28 (29) | 0/5 | 2/5 (40) | 1/3 (20) | 2/5 (40) | 2/5 | 1/5 (20) |
| **Hx of hypertension, n (%)** | 12/28 (43) | 2/5 (40) | 2/5 (40) | 2/3 (40) | 2/5 (40) | 2/5 | 2/5 (40) |
| **Hx of CAD, n (%)** | 12/28 (43) | 0/5 | 2/5 (40) | 0/3 | 5/5 (100) | 3/5 (60) | 2/5 (40) |
| **Hx of CABG, n (%)** | 5/28 (18) | 0/5 | 2/5 (40) | 0/3 | 3/5 (60) | 0/5 | 1/5 (20) |
| **Clinical cTnT, ng/L** | 80.5 (4.0- 147.25) | NA | 40.0 (20.0-725.0) | 16.0 (8.0-23.0) | 1,956.0 (1,250.0-3,380.0) | 80.0 (75.0-81.0) | 145.0 (132.0-148.0) |
| **Crea, mg/dL** | 2.2±3.0 | 0.83±0.14 | 1.5±0.6 | 0.9±0.08 | 1.0±0.4 | 0.8±0.2 | 7.9±3.0 |
| **eGFR, mL/min** | 58.8±38.0 | 100.2±19.2 | 40±16 | 85±6 | 61±22 | 87±17 | 9.4±6.2 |
| **Total Cholesterol, mg/dL** | 156.6±37.1 | 175.3±22.1 | 119.3±21.3 | 175.0±13.1 | 153.0±47.8 | 156.5±50.2 | 169±30 |
| **LDL, mg/dL** | 80.7±26.8 | 95.8 ± 9.7 | 58.3±10.5 | 87.3±14.0 | 98.0±22.8 | 72.0±47.1 | 67±18 |
| **NT-proBNP, pg/mL** | 2,256.5 (488.25-6,588.0) | NA | 6,155.0 (5,131.7-7,365.5) | 495.0 (340.5-1,516.5) | 1,300.5 (549.0-2,302.75) | 453.5 (412.75-663.5) | 123,528 (71,444-132,749) |
| **LVEF, %** | 50±18 | 64±5 | 22±11 | 61±1 | 51±14 | 58±8 | 50±19 |
| **IVS, mm** | 10±2 | 9±2 | 10±4 | 13.3±3 | 10±2 | 10±1 | 11±2 |
| **PWT, mm** | 10±2 | 8±0.5 | 9±4 | 13±2 | 10±2 | 9±1 | 11±2 |
| **Betablocker, n (%)** | 14/28 (50) | 1/5 (20) | 4/5 (80) | 2/3 (40) | 4/5 (80) | 3/5 (60) | 0/5 |
| **ACE, AT I, n (%)** | 6/28 (21) | 1/5 (20) | 2/5 (40) | 0/3 | 2/5 (40) | 1/5 (20) | 0/5 |
| **Diuretic, n (%)** | 8/28 (29) | 0/5 | 5/5 (100) | 1/3 (20) | 0/5 | 1/5 (20) | 1/5 |

**Table 1. Patient characteristics.** Where indicated values represent the number of patients per group and percentage, otherwise values represent average ± SD. For clinical cTnT and NT-proBNP values represent the median and interquartile range (25%-75%).

**Supplemental Figures**

**
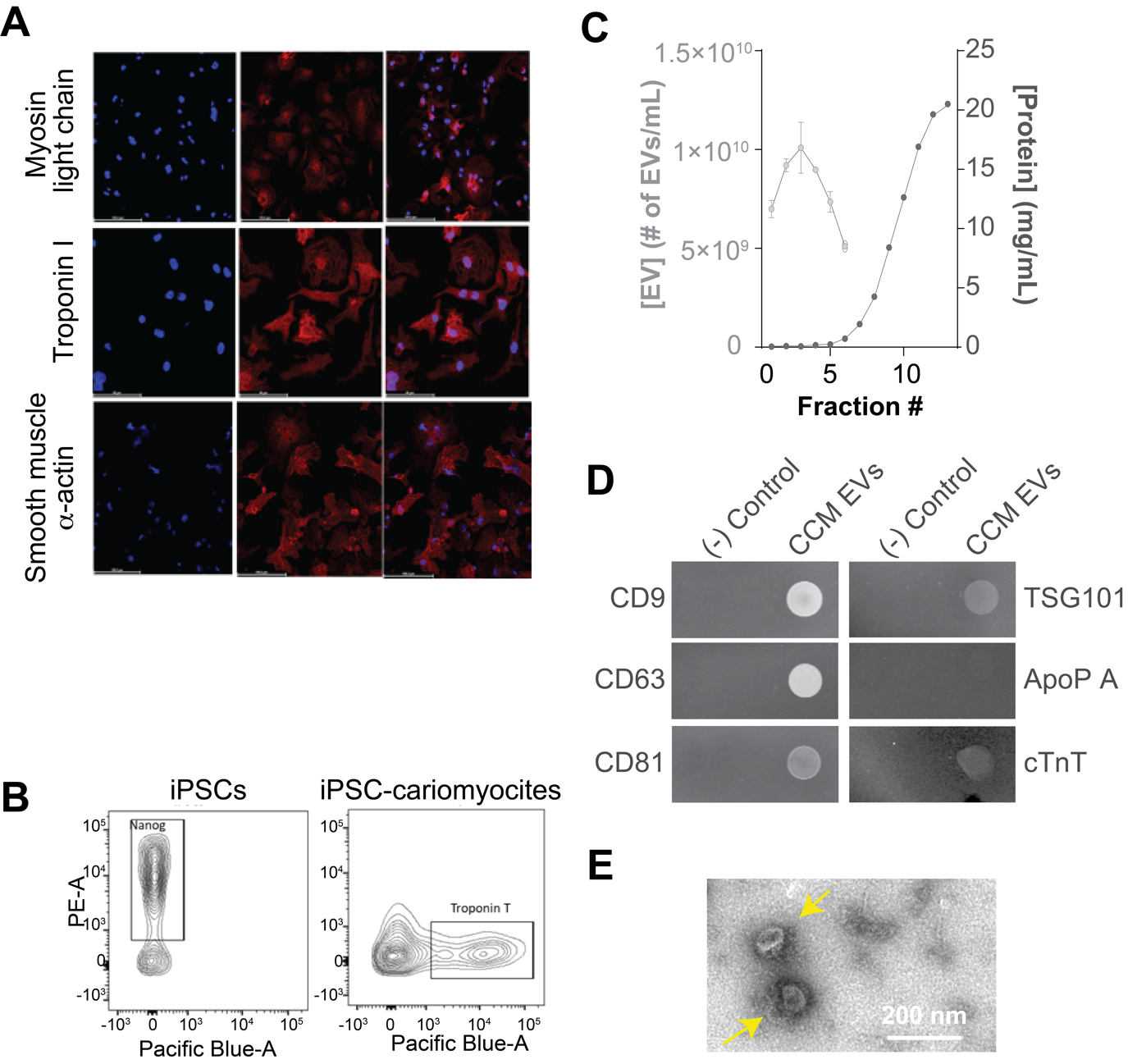
**

**Figure S1: iPSC-derived cardiomyocyte media EV characterization** (**A**) iPSCs differentiated into cardiomyocytes (CM), were stained with antibodies against myosin light chain, troponin I, and smooth muscle actin, and visualized under confocal microscope. iPSC-CMs were positive for Alexa-594. Cells were counter stained with Hoechst dye and overlay images were created. (**B**) Similarly, iPSCs and iPSC-derived cardiomyocytes were stained with antibodies against Nanog (PE) and Troponin T (Pacific blue A). The positive signals were recorded in a flow cytometer. (**C**) size exclusion chromatography (SEC) isolated EVs from CCM were characterized for EV concentration and protein content. Fraction collector fractions 1-4 had a high concentration of EVs and a low concentration of protein. These fractions were pooled for subsequent analysis. (**D**) Dot blots of EVs isolated from CCM or media alone using SEC; control media was processed in the same manner, including SEC purification, as media used for cell growth. CCM EVs were positive for EV markers (CD9, CD63, CD81, TSG101) and cTnT and negative for control Apo protein A. No signal was detected with control media. (**E**) Representative TEM image of EVs from CCM show intact EV morphology (EVs are indicated with arrows).

**
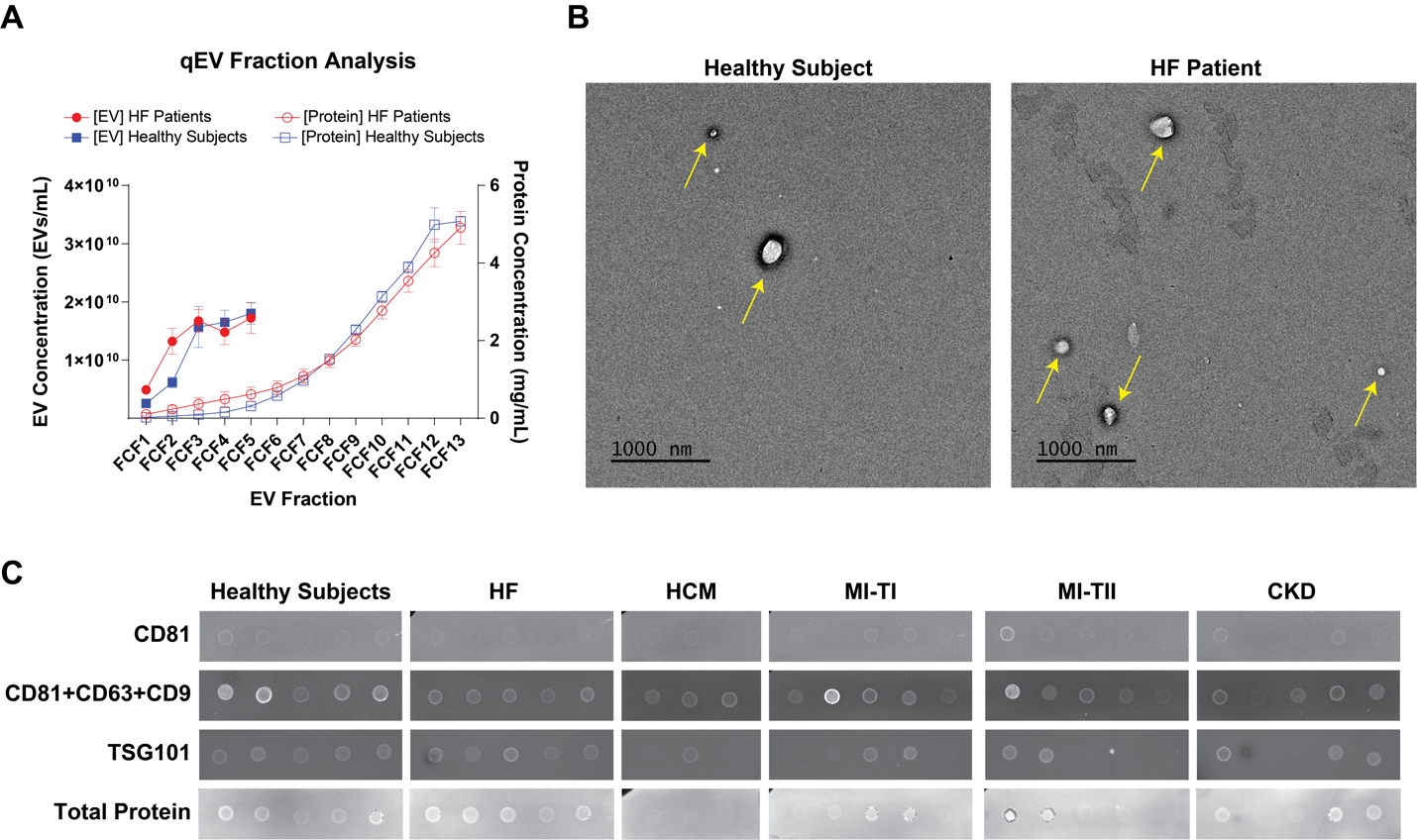
**

**Figure S2: Plasma EV characterization.** (**A**) EV and protein concentrations of healthy subjects (blue) and heart failure (HF) patients (red). (**B**) Representative TEM images of one healthy subject and one HF patient sample. Yellow arrows point to EVs. (**C**) Both membrane EV markers (tetraspanins CD9, CD63, CD81) and luminal EV marker (TSG101) are present in EVs. Dot blots show the expression of CD81, all canonical tetraspanins (CD81, CD63, and CD9), TSG101, and total protein (determined using Sypro Ruby). Human subjects had a unique content of EV markers. While CD81 content between patients was highly variable agnostic of disease classification, a combination of CD9, CD63, and CD81 was more uniform in expression. TSG101 content had moderate variability between patients and largely scaled with total protein content.


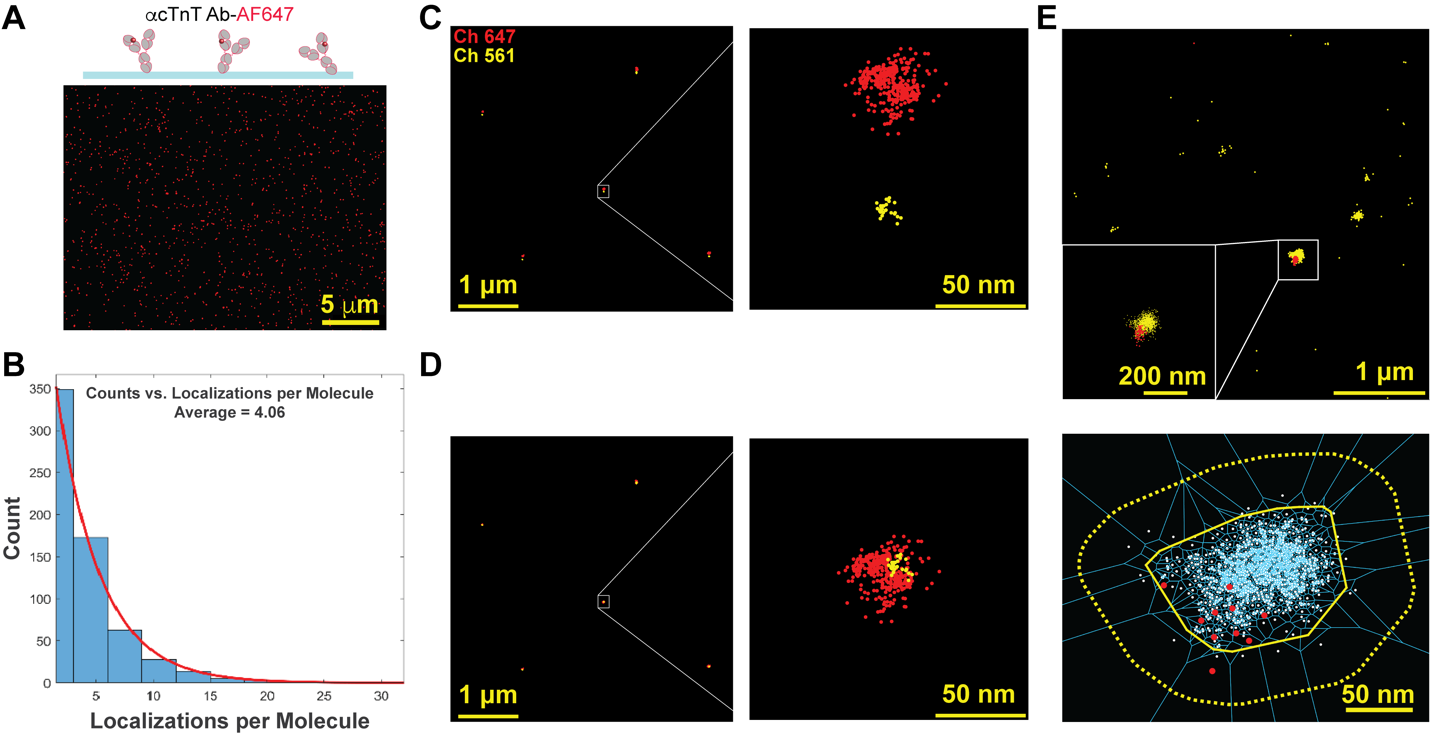


**Figure S3: qSMLM analysis**. (**A**) A representative region of cTnT Ab-AF647 coated surfaces. There is a sparse, evenly distributed fluorescent signal. (**B**) The average number of localizations from individual Alexa Fluor 647 labeled cTnT Ab (alpha) from three independent experiments, 20 FOVs, was 4. (**C**) Beads were localized in two channels and the warp polynomial equation was fit to reduce the distance between bead centers detected in multiple channels (**D**). The resulting polynomial warping equations were applied to all subsequent images, reducing the effect of chromatic aberration. (**E**) **Top**, Raw images of EVs were detected with CF568 (yellow) and cTnT Ab-AF647 (red). **Bottom,** Localizations in 561 nm are tessellated to create polygon areas, analysis thresholds in Methods refer to polygon areas to define EV borders. Once EV borders are defined (solid yellow line) and localizations in the 647 nm channel are processed (red dots), any 647 nm channel localizations within 50 nm of the EV border (dashed yellow line) are counted (see Methods).

**
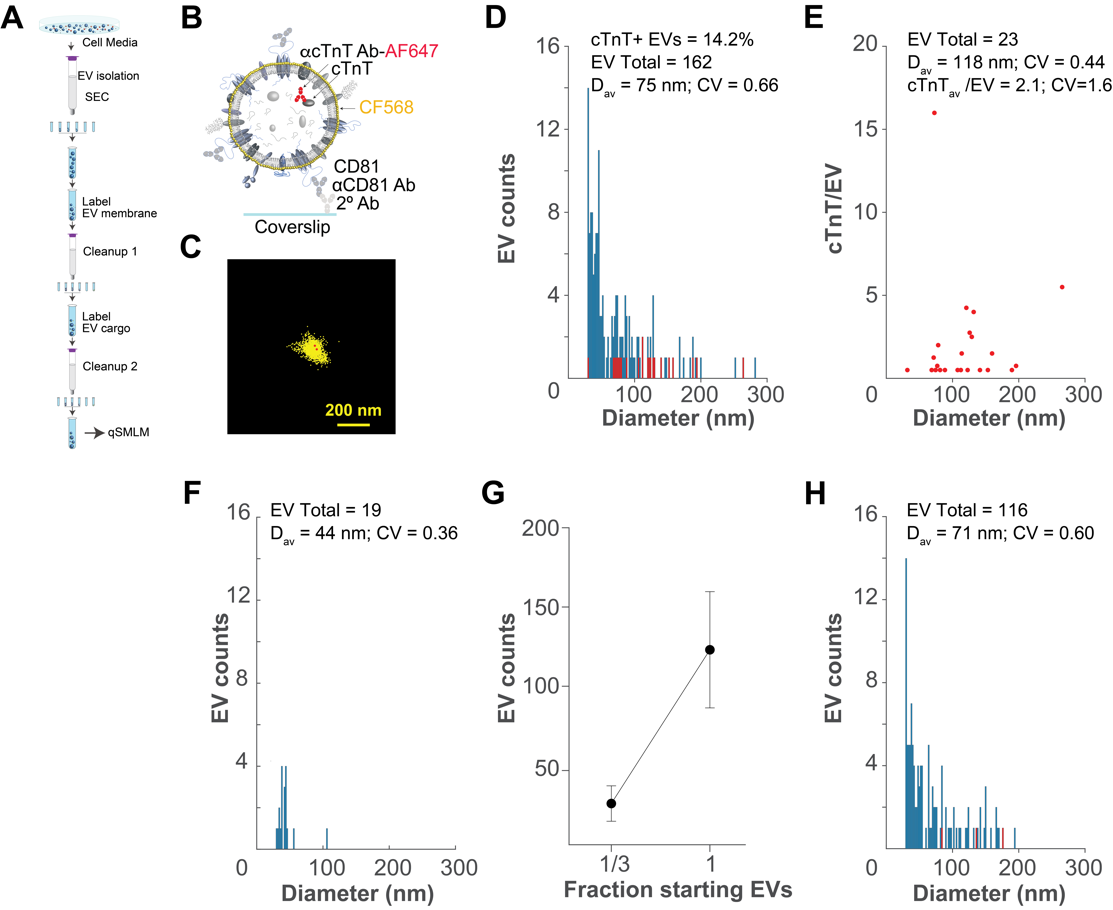
**

**Figure S4. Characterization of EVs from CCM.** (**A**) Scheme of the experiment. EVs from iPSC-derived cardiomyocyte cell media were purified using size exclusion chromatography (SEC). For qSMLM, the EV membrane was fluorescently labeled with CF568 dye (yellow), and excess dye was removed. Under gentle permeabilization conditions, luminal cTnT in EVs was labeled with AF647 tagged Ab (to detect cTnT, in red) and membrane CD81 in EVs was labeled with unmodified Ab (to affinity isolate EVs). After removal of excess Abs, CD81 enriched EVs were affinity isolated onto coverslips coated with anti-mouse secondary Ab. (**B**) Scheme of the labeled EV: membrane was covalently labeled with CF568 dye (yellow), cTnT was affinity labeled with AF647 tagged Ab and CD81 was affinity labeled with unmodified Ab (to affinity isolate EVs onto coverslips coated with secondary anti-mouse Ab). (**C**) qSMLM image of CD81-enriched EVs: CF568 localizations in yellow (detection of EV membrane) and cTnT Ab-AF647 localizations in red (detection of cTnT). (**D**) qSMLM quantification of CD81-enriched EVs from CCM. CF568 localizations were used to identify all CD81-enriched EVs and determine their size while cTnT Ab-AF647 localizations were used to identify a subset of cTnT-positive EVs. Size histogram of all CD81-enriched EVs is shown in blue. 162 EVs were detected with average diameter of 75 nm with a CV of 0.66. Size histogram of CD81-enriched, cTnT-positive EVs is shown in red. (**E**) Distribution of size (x-axis) and detected cTnT molecules/EV (y-axis) for CD81-enriched, cTnT-positive EVs; each EV is represented with a red dot. These EVs had average diameter of 118 nm with a CV of 0.44 and average of 2.1 detected cTnT molecules per EV with a CV of 1.6. (**F**) Few EVs were isolated on the control αRabbit-antibody coated surface. (**G**) Number of detected EVs correlated with the amount of EVs incubated onto coverslips. Error bars represent SEM. (**H**) Size histogram of CD81-enriched EVs from CCM processed without Tween-20 permeabilization is shown in blue. 116 EVs were detected with an average diameter of 71 nm with a CV of 0.60. Only three signals (red) were detected in the 647 nm channel when Tween-20 was not used to permeabilize EVs. For **D-H**, n=3, 15 fields of view were analyzed.
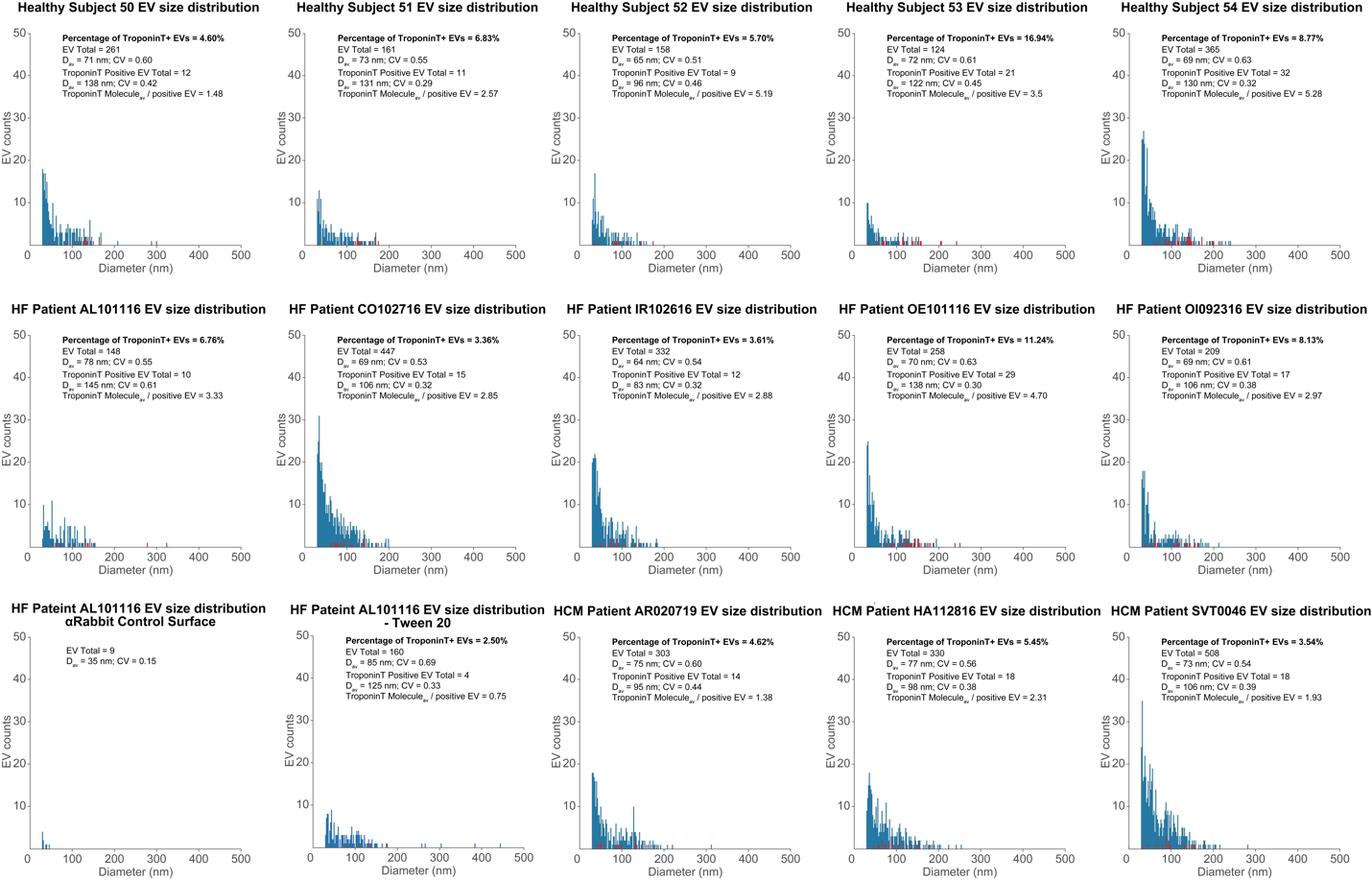


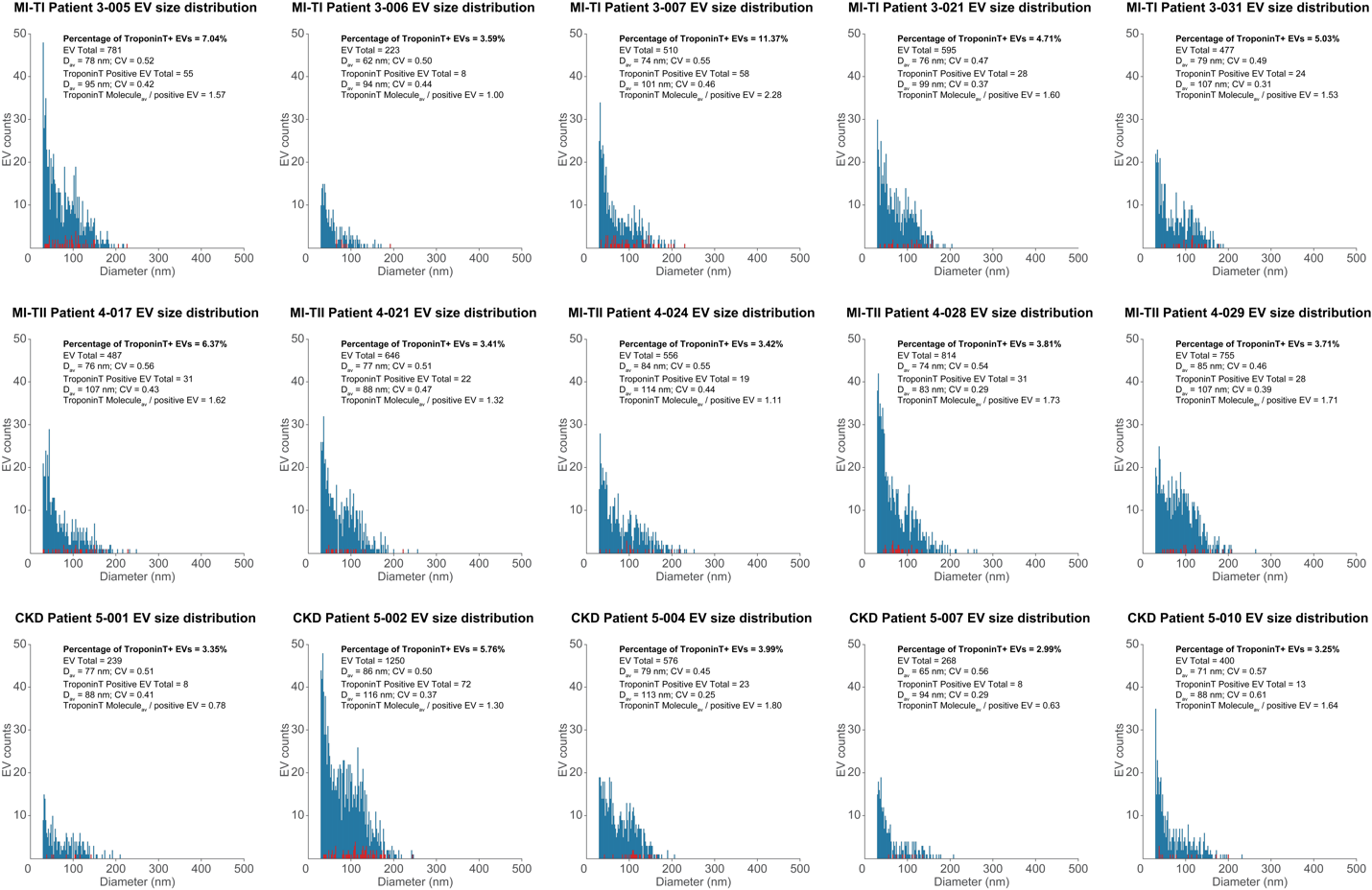


**Figure S5: Size heterogeneity of tetraspanin-enriched EVs from patient plasma**. Individual histograms of total tetraspanin-enriched EV populations (blue) for heart failure (HF) patients, hypertrophic cardiomyopathy (HCM) patients, myocardial infarction type I (MIT-I) patients, myocardial infarction type II (MIT-II) patients, and chronic kidney disease (CKD) patients. cTnT-positive EVs are indicated in red. EV characteristics include the percentage of cTnT-positive EVs, total number of EVs, diameter of tetraspanin-enriched EVs, and diameter of tetraspanin-enriched, cTnT-positive EVs. αRabbit antibody surface control and negative Tween-20 control are shown for an HF patient.


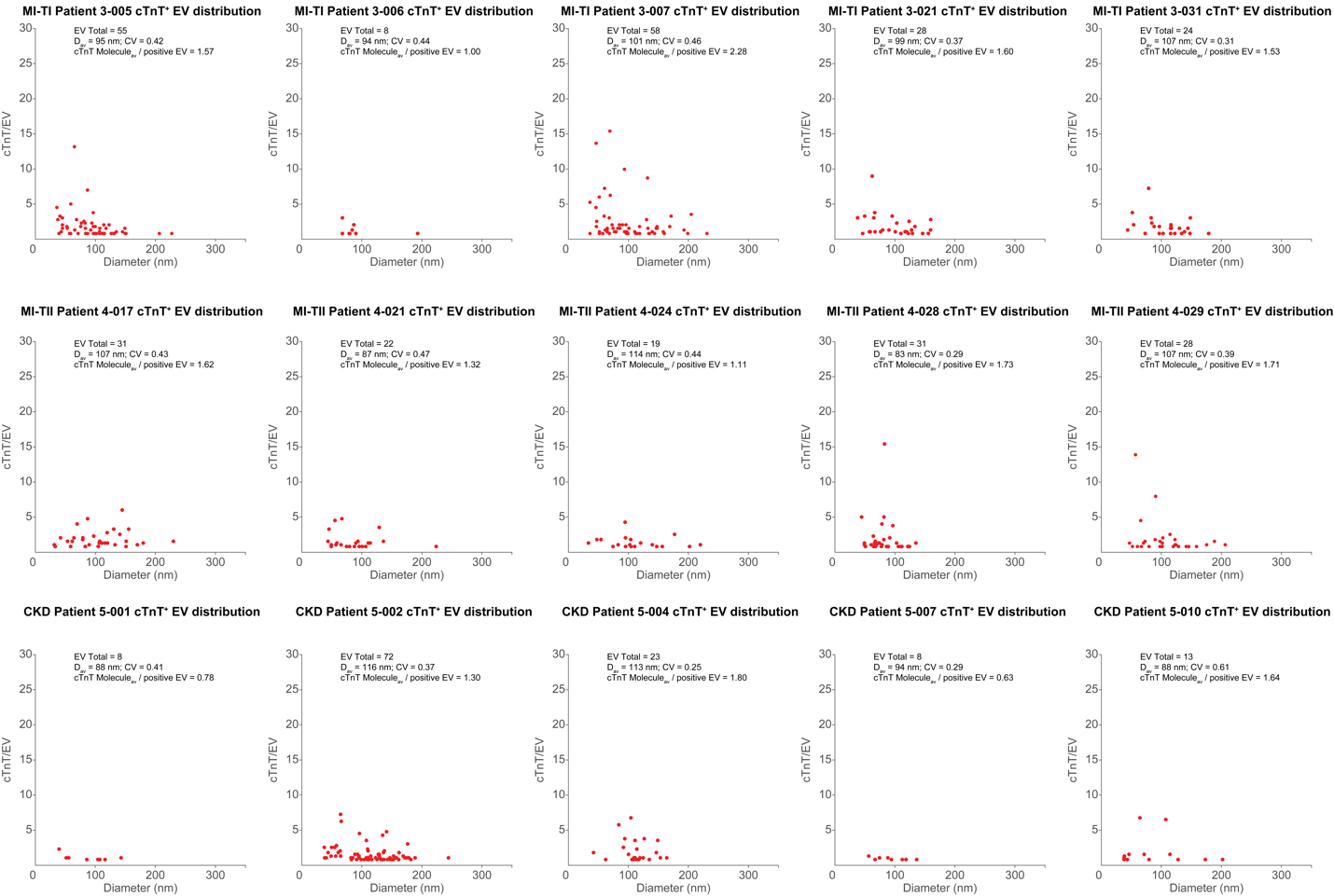

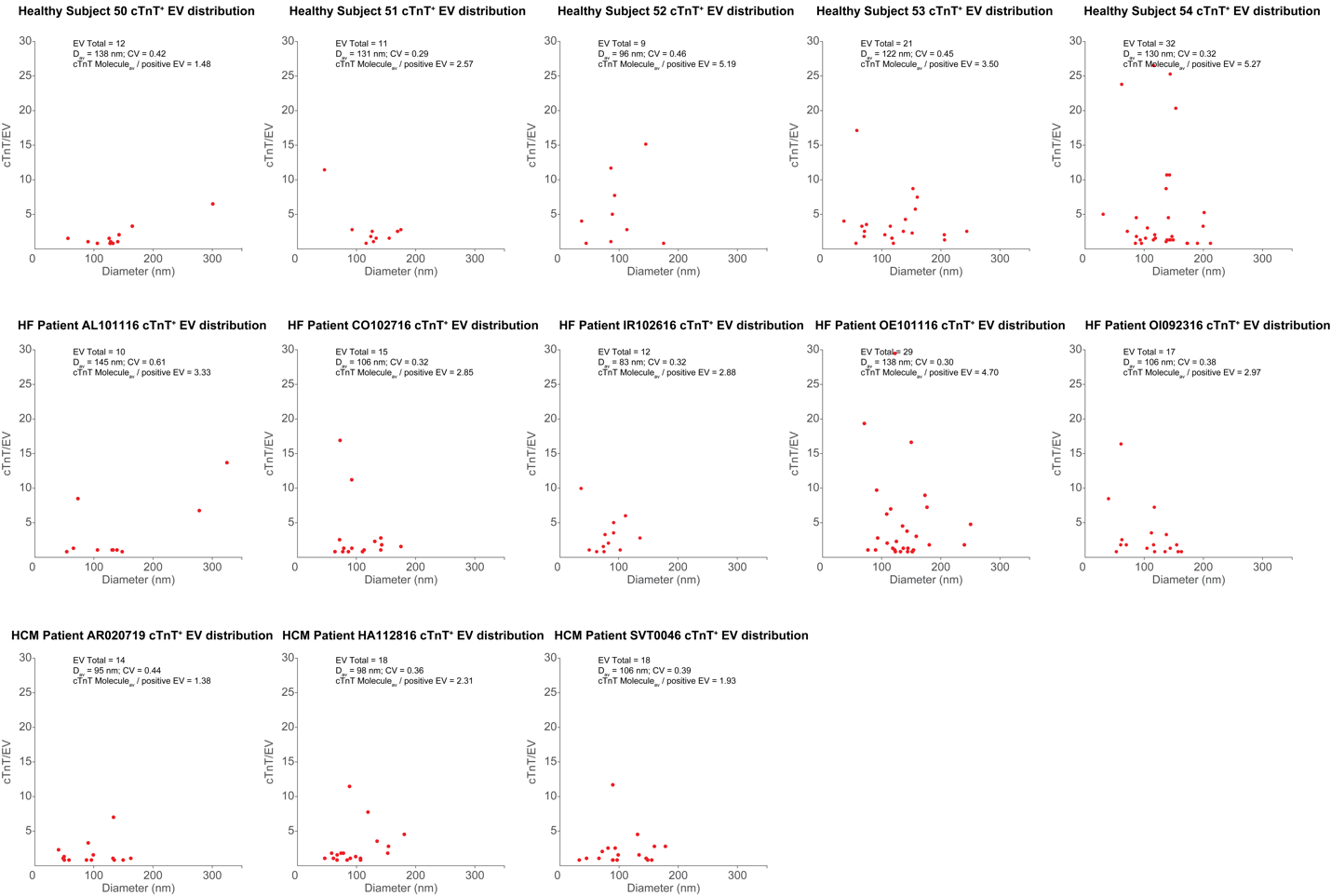


**Figure S6: Heterogeneity of tetraspanin-enriched, cTnT-positive EVs from patient plasma**. Individual 2D scatter plots of cTnT-positive EVs for heart failure (HF) patients, hypertrophic cardiomyopathy (HCM) patients, myocardial infarction type I (MIT-I) patients, myocardial infarction type II (MIT-II) patients, and chronic kidney disease (CKD) patients. Scatter plots indicate two characteristics for detected EVs (red dot): individual EV size (x-axis) and amount of detected cTnT associated with that EV (y-axis). Average EV characteristics include the number of cTnT-positive EVs (EV Total), diameter (D_av_) and coefficient of variation (CV) of cTnT-positive EVs, and average detected cTnT per EVs (cTnT molecule_av_ /positive EV).


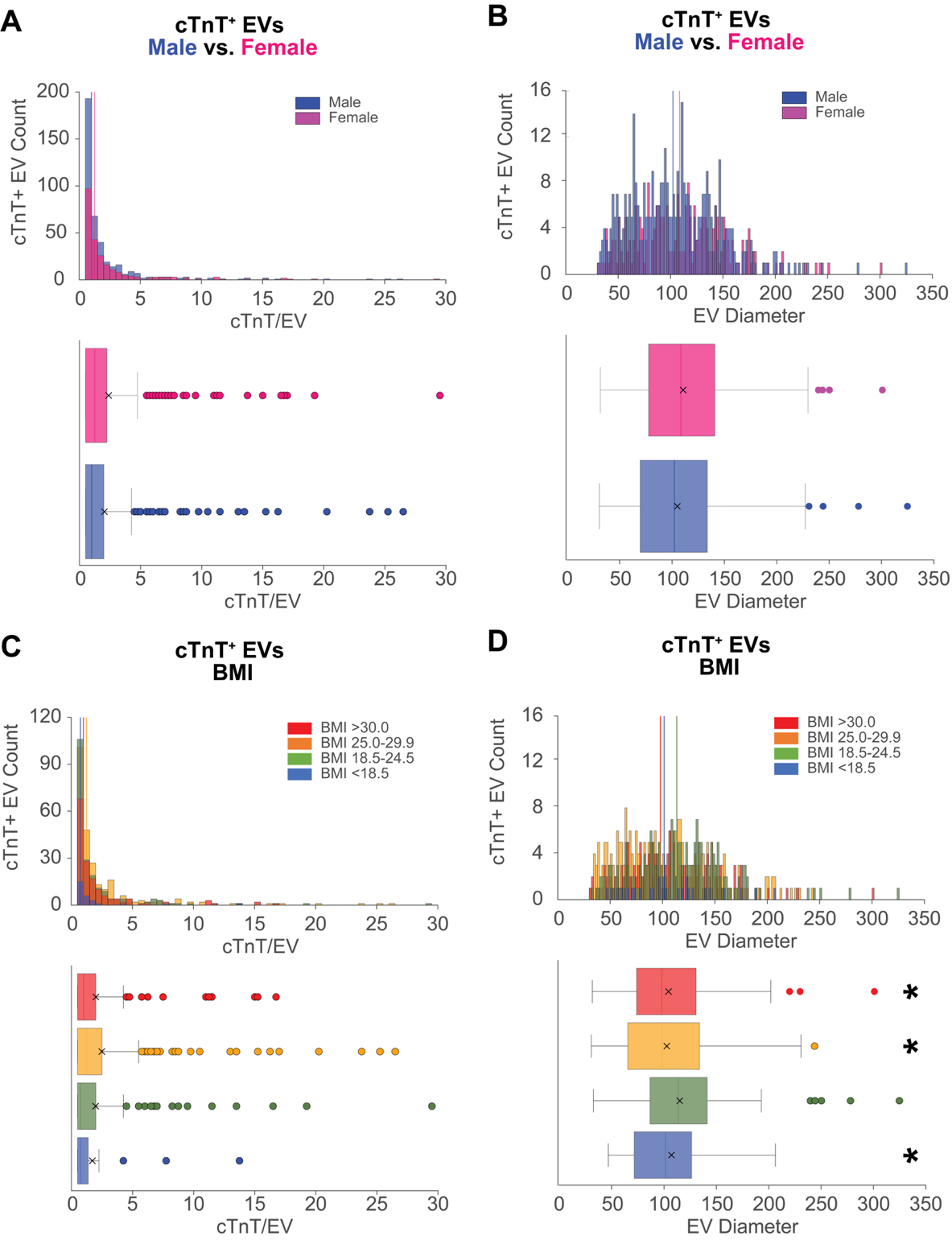


**Figure S7: Characterization of tetraspanin-enriched, cTnT-positive EVs across subject characteristics**. (**A, B**) EVs from male and female subjects were grouped. Histograms of distributions for detected cTnT/EV (**A**) and EV size (**B**) are on top, while corresponding box plots are on the bottom. (**C, D**) EVs from subjects with different BMI values were grouped. Histograms of distributions for detected cTnT/EV (**C**) and EV size (**D**) are on top, while corresponding box plots are on the bottom. Of note, a single patient had BMI<18.5. Box plots represent the interquartile range, median (center line), and mean (indicated with x); dots indicate EVs beyond 1.5 times the interquartile range.
